# Modelling the onset of senescence at the G1/S cell cycle checkpoint

**DOI:** 10.1186/1471-2164-15-S7-S7

**Published:** 2014-10-27

**Authors:** José CM Mombach, Cristhian A Bugs, Claudine Chaouiya

**Affiliations:** 1 Universidade Federal de Santa Maria, Santa Maria, RS, Brazil; 2 Instituto Gulbenkian de Ciência, Oeiras, Portugal; 3 Universidade Federal do Pampa, São Gabriel, RS, Brazil

**Keywords:** Signalling network, Logical modelling, Senescence, DNA-damage, Cell fate, Cell cycle checkpoint

## Abstract

**Background:**

DNA damage (single or double-strand breaks) triggers adapted cellular responses. These responses are elicited through signalling pathways, which activate cell cycle checkpoints and basically lead to three cellular fates: cycle arrest promoting DNA repair, senescence (permanent arrest) or cell death. Cellular senescence is known for having a tumour-suppressive function and its regulation arouses a growing scientific interest. Here, we advance a qualitative model covering DNA damage response pathways, focusing on G1/S checkpoint enforcement, supposedly more sensitive to arrest than G2/M checkpoint.

**Results:**

We define a discrete, logical model encompassing ATM (ataxia telangiectasia mutated) and ATR (ATM and Rad3-related) pathways activation upon DNA damage, as well as G1/S checkpoint main components. It also includes the stress responsive protein p38MAPK (mitogen-activated protein kinase 14) known to be involved in the regulation of senescence. The model has four outcomes that convey alternative cell fates: proliferation, (transient) cell cycle arrest, apoptosis and senescence. Different levels of DNA damage are considered, defined by distinct combinations of single and double-strand breaks. Each leads to a single stable state denoting the cell fate adopted upon this specific damage. A range of model perturbations corresponding to gene loss-of-function or gain-of-function is compared to experimental mutations.

**Conclusions:**

As a step towards an integrative model of DNA-damage response pathways to better cover the onset of senescence, our model focuses on G1/S checkpoint enforcement. This model qualitatively agrees with most experimental observations, including experiments involving mutations. Furthermore, it provides some predictions.

## Background

Numerous checkpoints ensure the correct progression along the phases (G0, G1, S, G2, M) of the eukaryotic cell cycle [1]. DNA damage response pathways activate checkpoints to arrest the cell cycle transiently, promoting DNA repair, or permanently, inducing senescence or cell death [2]. DNA damage consists of DNA single-strand breaks (SSB) and double-strand breaks (DSB) that present a threat to structural chromosome stability and are thus the main inducers of DNA damage response [2]. This response to specific DNA damage is an evolutionary program that prevents the propagation of incorrect genomic information. Mammalian senescence, a less understood complex phenotype, is associated to aging and tumorigenesis [3,4]. The secretory phenotype associated with the senescent state includes growth factors that affect cells and tissues by activating membrane receptors whose deregulation is responsible for numerous pathologies, including cancer [5,6].

Upon DNA damage, the mechanisms driving the decision between these different cell fates are still unclear and, partly due to a manifest medical impact, they are the subject of high interest (e.g., [7,8]). In particular, how cells are induced to senescence upon DNA damage attracted a lot of attention, this phenotype being associated to tumour suppression [9]. Here, we propose to resort to a qualitative modelling approach to investigate these mechanisms.

A variety of modelling frameworks offer complementary tools to integrate current knowledge in the form of computational models that provide insights into biological processes (e.g., [10-13]). Recently, several groups have approached cell fate decision from different modelling perspectives, from differential equations to discrete models.

Among other components, the tumour suppressor p53 protein triggers cellular programs that lead to different fates: transient arrest followed by cell cycle re-entry upon damage repair, permanent cell cycle arrest (senescence) or, if the damage is irreparable, initiation of cell death program (apoptosis). To investigate the decision between G1 arrest and apoptosis, Zhang *et al*. proposed a modular model of the p53 network, using ordinary differential equations [9]. The model defined by Iwamoto *et al*. also comprises p53 together with cell cycle regulation allowing to investigate the impact of DNA damage intensities on cell cycle progression [14]. Purvis and co-workers proposed a model based on delayed differential equations to further study how p53 dynamics influences the decision between apoptosis and senescence [15].

In contrast to these continuous models, discrete, logical formalisms were used to tackle the study of networks implicated in cell cycle control (see [16] for a review) as well as in cell fate decision [17-20]. Calzone *et al*. [19] advanced with a model of cell fate decision with death and survival receptors as input signals, downstream pathways and three cellular outcomes: survival, apoptosis and necrosis. Also relying on a logical approach, Poltz and Naumann devised a model to study the inflammation contribution to DNA damage response leading to cell cycle arrest or apoptosis [18]. Finally, the recent model from Grieco *et al*. accounts for the influence of the stress responsive mitogen-activated protein kinase pathway (MAPK) on cancer cell fate decision [20].

Searching the literature and partly building on the network of human G1/S checkpoint activation [2], we propose a logical model of cell fate decision upon DNA damage, including proliferation, transient arrest for DNA repair, apoptosis and, notably, senescence. This precursory model extends the regulatory pathway of G1/S checkpoint activation with the inclusion of the stress responsive protein p38MAPK (mitogen-activated protein kinase 14) that plays an important role in senescence [7,8,21].

The paper is organised as follows. After a brief overview of main biological facts, our model is defined and analysed in the Results section. The Conclusion section is devoted to further discussion of the model properties and includes future work. The logical modelling framework is described in the Methods section.

## Results and discussion

**Figure 1:**
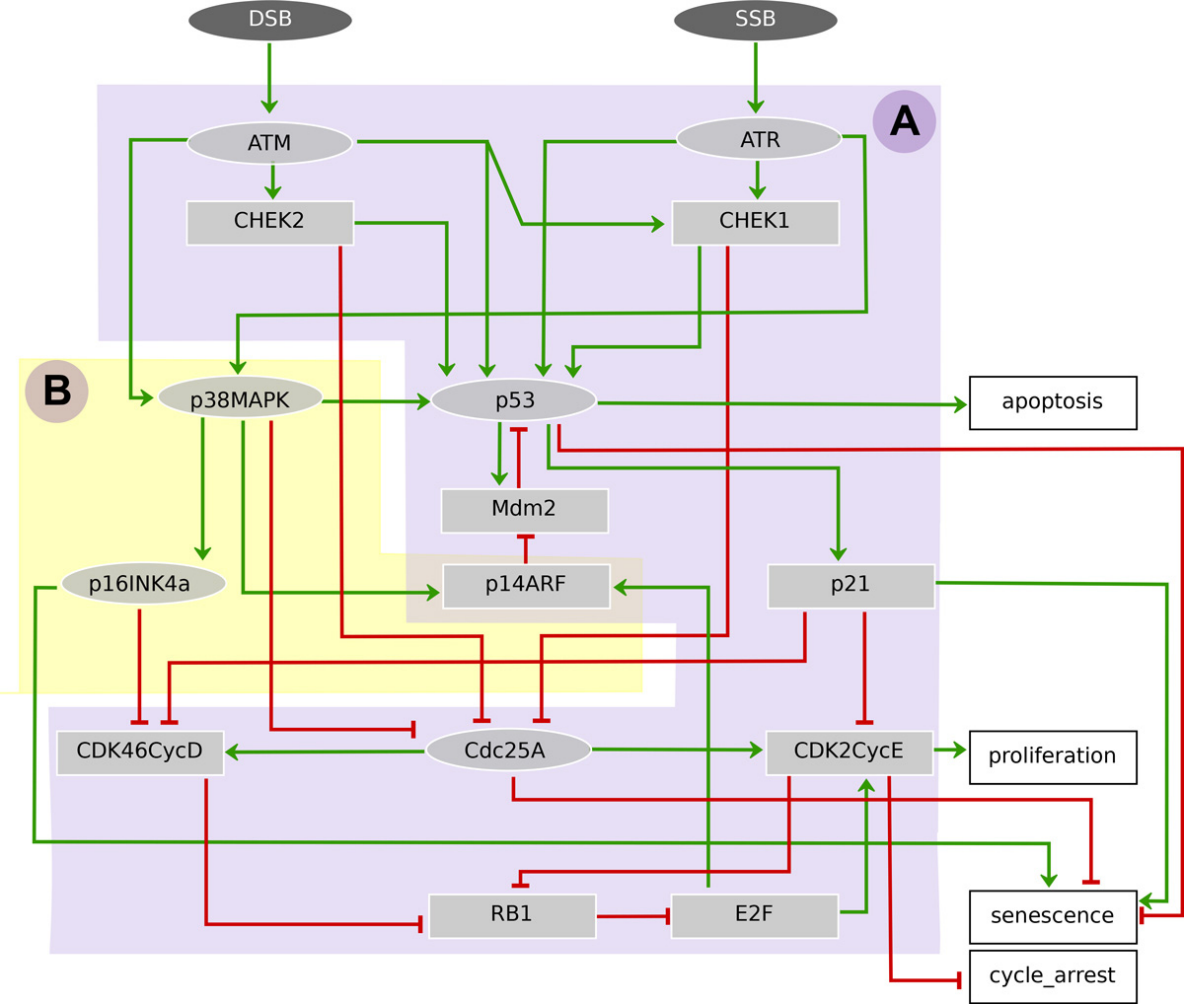
**Regulatory network of cell-fate decision upon DNA damage involving (A) the G1/S checkpoint arrest network and (B) the senescence regulatory pathway**. Rectangular nodes indicate Boolean components, whereas ellipses represent multi-valued components; green arrows represent activations and red bar arrows inhibitions. Input nodes (dark grey ellipses) SSB and DSB stand for single and double DNA-strand breaks respectively. Output nodes (white rectangles) correspond to the four different outcomes (cell fates). Internal nodes embody the main regulators of the fate decision.

We briefly describe biological facts at the core of the regulatory network of Figure 1, the corresponding logical model being then thoroughly defined. This section ends with the model analysis, in terms of cell fates for the wild type situation and a range of perturbations.

In what follows, we first introduce well-established molecular processes responsible for G1/S checkpoint and apoptosis activation. Then, as the main novelty of our work, we describe additional players involved in regulating human cellular senescence through G1/S checkpoint activation.

### G1/S checkpoint and apoptosis (Figure 1-A)

Presence of DNA damage activates checkpoints, halting progression of the cell cycle [2,22]. Arrest for repair, apoptosis or senescence can be triggered both at G1/S and G2/M checkpoints. In the case of senescence-induced DNA damage, it was suggested that G1/S checkpoint is more sensitive and that a single DSB can induce arrest, while a larger number is required to activate G2/M checkpoint [23]. Molecular machineries sense DNA strand breaks and activate the kinases ataxia telangiectasia mutated (ATM) and Rad3-related (ATR) and consequently downstream pathways involving the activation of p53 [22,23]. ATM is activated by DSB while ATR is activated by either SSB or DSB, and they both trigger a cascade of phosphorylations accounting for most of the interactions represented in Figure 1[2,8]. ATM phosphorylates the kinases checkpoint kinase 2 (CHEK2) while ATR phosphorylates the kinases checkpoint kinase 1 (CHEK1). CHEK1 and CHEK2 initiate the cell cycle arrest by phosphorylating cell division cycle 25A protein (CDC25A) that prevents the dephosphorylation of the protein complexes cyclin-dependent kinase 4, 6 and cyclin D (Cdk4/6-Cyclin-D) and cyclin-dependent kinase 2 and cyclin E (Cdk2/Cyclin-E). This also prevents the phosphorylation of retinoblastoma 1 protein (RB1) and the release of E2F transcription factors that induce the expression of genes required for the cell to enter S phase [2]. ATR, ATM, CHEK1 and CHEK2 phosphorylate p53 that mediates the maintenance of G1/S arrest through the activation of cyclin-dependent kinase inhibitor 1A (p21), which in turns inhibits Cdk4/6-Cyclin-D and Cdk2/Cyclin-E [24,25]. Upon DNA repair, the complex Cdk2/Cyclin-E is activated and drives the cell from G1 to S phase.

Decision between growth arrest and apoptosis is mediated through a pivotal threshold mechanism related to the activation level of p53 that, when surpassed, triggers apoptosis at G1/S checkpoint [26]. The locus CDKN2A contributes to cell cycle regulation through its two products: p14ARF (the alternate reading frame product), which promotes p53 and p16^INK4a ^involved in senescence (see below) [4,27]. The regulatory module involving E3 ubiquitin protein ligase homolog (Mdm2) and p14ARF tightly controls p53; Mdm2 promotes p53 degradation, while Mdm2 is sequestered by p14ARF to decrease p53 degradation [27]. In our model, apoptosis is oversimplified; it is activated when p53 reaches its highest level.

### Senescence (Figure 1-B)

The regulation of the senescent state, although less understood, is known to be associated with the activation in several cell types of the p53-p21 pathway (that stabilizes the arrest, see above) and of the p16^INK4a^-RB1 pathway [4,7,21].

Cyclin-dependent kinase inhibitor 2A (p16^INK4a^) contributes along with p53 to block cancer progression since it inhibits the complex Cdk4/6-Cyclin-D required for proliferation. The overexpression of p16^INK4a ^can induce a senescent arrest in several human cell types [4,7]. The exact mechanisms of regulation of p16^INK4a ^(a product of CDKN2A locus) are still unclear, however p38MAPK is involved somehow [21,28,29].

p38MAPK is a component of the ATM/ATR dependent MAPK stress responsive pathway. Besides regulating the locus CDKN2A, p38MAPK activates p53 leading to arrest or apoptosis and inhibits Cdc25A required for proliferation [8,30,31]. Overexpression of p38MAPK induces senescence even in the absence of DNA damage [32].

### Logical model

Relying on the aforementioned biological evidences, and focusing on the inclusion of senescence as an additional outcome of the checkpoint arrest, we delineate a logical version of the regulation of the G1/S checkpoint. A description of the modelling framework is provided in the Methods section.

The network of Figure 1 should be seen as a generic wiring since senescence requires the involvement of both p16^INK4a^-RB1 and p53-p21 pathways in several cell types [4]. Table 1 includes a brief documentation of the network nodes. The logical rules governing the states of the nodes briefly described below are given in Table 2.

**Table 1:** Brief molecular description of the model components.

Node	Description
SSB	Single strand break: 0 (no break), (1) reparable and (2) irreparable SSB

DSB	Double strand break: 0 (no break), (1) reparable and (2) irreparable DSB

ATR	Ataxia telangiectasia and Rad3 related protein

ATM	Ataxia telangiectasia mutated protein

CHEK2	Checkpoint kinase 2 protein

CHEK1	Checkpoint kinase 1 protein

p14ARF	Alternate reading frame (ARF) protein (from CDKN2A locus)

p16^INK4a^	Cyclin-dependent kinase inhibitor 2A protein (from CDKN2A locus)

p38MAPK	Mitogen activated protein kinase 14 protein

Mdm2	E3 ubiquitin protein ligase homolog protein

p21	Cyclin-dependent kinase inhibitor 1A protein

p53	Tumor supressor protein p53 protein

CDC25A	Cell division cycle 25A protein

E2F	E2F transcription factor family of proteins (E2F1, E2F2, E2F3)

RB1	Retinoblastoma 1 protein

CDK46CycD	Protein complex: Cyclin-dependent kinase 4, 6 and Cyclin D

CDK2CycE	Protein complex: Cyclin-dependent kinase 2 and Cyclin E

**Table 2:** Logical rules associated with the regulatory network of Fig 1 and interpretation of the multi-levels.

Node	Rule / level interpretation	
ATM	1: DSB = 1	Low level of DSB signal
	
	2: DSB = 2	High level of DSB signal

ATR	1: SSB = 1	Low level of SSB signal
	
	2: SSB = 2	High level of SSB signal

CHEK2	1: ATM = 2	

CHEK1	1: ATR = 2 *OR *ATM = 2	

p14ARF	1: p38MAPK = 2 *OR *E2F	

p38MAPK	1: (ATM = 1 *OR *ATR = 1-2) *AND NOT*(ATM = 2)	Activated p38MAPK pathway (leading to cycle arrest)
	
	2: ATM = 2 *AND NOT*(ATR = 2)	Middle level of p38MAPK pathway activation (leading to senescence)
	
	3: ATM = 2 *AND *ATR = 2	Highest level of p38MAPK pathway activation (leading to apoptosis)

Mdm2	1: p53 = 1 *AND NOT*(p14ARF)	

p16INK4A	1: p38MAPK = 1-2	Activated p16^INK4A^
	
	2: p38MAPK = 3	p16 ^INK4A ^upregulation

p21	1: p53 = 1	

p53	1: Mdm2 = 1 *AND *(p38MAPK = 3 *OR *ATR = 1-2 *OR *ATM = 1-2 *OR *CHEK1 = 1 *OR *CHEK2 = 1)	Activated p53 (no accumulation)
	
	2: *NOT*(Mdm2 = 1) *AND *(p38MAPK = 3 *OR *ATR = 1-2 *OR *ATM = 1-2 *OR *CHEK1 = 1 *OR *CHEK2 = 1)	p53 accumulation leading to apoptosis

CDC25A	1: (p38MAPK = 1-3 *OR *CHEK2 = 1 *OR *CHEK1 = 1) AND NOT(p38MAPK = 1-3 *AND *CHEK2 = 1 *AND *CHEK1 = 1)	Low concentration of active Cdc25A (i.e. non-phosphorylated)
	
	2: *NOT*(p38MAPK = 1-3) *AND NOT*(CHEK2 = 1) *AND NOT*(CHEK1 = 1)	High concentration of active Cdc25A

E2F	1: *NOT*(RB1 = 1)	

RB1	1: *NOT*(CDK46CycDc = 1) *AND NOT*(CDK2CycEc =1)	Dephosphorylated RB1 bound to E2F

CDK46CycD	1: Cdc25A = 1 *AND NOT*(p16INK4a = 1-2) *AND NOT*(p21 = 1)	

CDK2CycE	1: *NOT*(p21 = 1) *AND *Cdc25A = 2 *AND *E2F = 1	

apoptosis	1: p53 = 2	

proliferation	1: CDK2CycEc = 1	

senescence	1: (p16INK4a = 1 *AND *p21 = 1 *AND NOT*(Cdc25A = 1-2) *AND NOT*(p53 = 2)) *OR *(p16INK4a = 2 *AND *p21 = 1 *AND NOT*(Cdc25A = 2) *AND NOT*(p53 = 2))	

cyclearrest	1: *NOT*(CDK2CycEc = 1)	

Input components (DSB and SSB) are constant and thus do not appear in this table. Rules are defined using the logical connectors *AND, OR *and *NOT*. For each node, they specify its target value, depending on the state of its regulators. Note that 0 is the default value (selected whenever none of the conditions defined for other values are verified).

SSB and DSB, the two input nodes of the network, take three values corresponding to damage intensities: 0 for no damage, 1 for a reparable damage and 2 for an irreparable damage. SSB and DSB levels determine the ATR and ATM levels, respectively, which in turn activate CHEK2, CHEK1, p38MAPK and p53.

The criterion for the activation of the 'proliferation' node (representing no arrest and transition to the S phase) is the activation of CDK2CycE. The 'cycle_arrest' node, denoting a transient arrest for repair, is ON in the absence of CDK2CycE.

Components p53 and p38MAPK have 3 and 4 levels, respectively, and play a central role in the network processing. The presence of intermediary DNA damage induces p53 to its intermediary level (p53 = 1) that is involved in several fates. To trigger apoptosis, p53 must reach its highest value (p53 = 2) [26]. In the model, this only occurs in case of fully irreparable DNA damage: DSB=SSB = 2.

p38MAPK affects cell fate decision through its interactions with p53, p16^INK4a ^and p14ARF. ATM has a stronger positive influence on p38MAPK than ATR [30]. Its first positive level (1) is reached when ATR is present or when ATM is present but not at its highest level. p38MAPK is at level 2 when ATM is at its maximum level but not ATR. When both ATM and ATR are at their maximum levels, *i.e*. in the case of fully irreparable DNA damage, p38MAPK reaches its highest level (3).

As previously mentioned, 'senescence' is regulated simultaneously by the p53-p21 and p16^INK4a^-RB1 pathways and corresponds to a permanent arrest where proteins involved in cell cycle progression are inhibited, especially CDC25A. Therefore, we consider that the activation of the fate 'senescence' requires both positive influences from p21 and p16^INK4a^, the absence of CDC25A and p53 not at its highest level. However, in the presence of CDC25A, senescence is activated provided p16^INK4a ^is at its highest level 2. Such a p16^INK4a ^significant overexpression, has been experimentally observed [33]. It was suggested that CHEK1 and CHEK2 may not have redundant roles in CDC25A regulation [34]. Accordingly, CDC25A has 3 levels; its 'full' inactivation being achieved only in the presence of CHEK1, CHEK2 and p38MAPK. In what follows, we analyse the model outcomes, given by its stable states, for the wild-type situation and a range of relevant perturbations.

### Model stable states: wild type case

**Figure 2:**
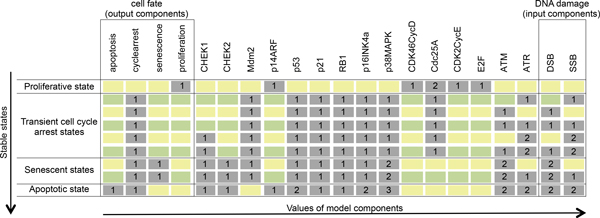
**Model stable states for the wild-type situation**. Each line corresponds to a stable state characterised by the values of the model components (listed in the columns). Each combination of SSB and DSB values among the 9 possible combinations (2 right-most columns) leads to a unique stable state; the corresponding cell fate is defined by the values of the output components (4 left-most columns).

Figure 2 displays the stable states of the model, in which all component values are maintained (see Methods section). Each type of DNA damage, described by the combination of the different levels of the input nodes DSB and SSB, defines a unique stable state, in which the values of the output nodes embody the cell fates ('proliferation', 'apoptosis', 'senescence', 'cycle_arrest'). In the absence of DNA damage, the outcome is 'proliferation' and in this stable state, the only active components are those involved in cell cycle progression. In the case of reparable damage, described by SSB or DSB = 1, the model outcome is 'cycle_arrest', interpreted as a transient arrest for repair that requires the inhibition of CDK2CycE.

The node 'senescence' is activated when DSB = 2 and SSB<2 as irreparable SSB does not induce senescence [4,23]. When activated, 'senescence' is accompanied by 'cycle_arrest'. For SSB=DSB = 2, 'apoptosis' is activated by the high level of p53 (and accompanied by 'cycle_arrest').

Summarising, the stable states of our model, in the wild type situation, are consistent: no damage leads to proliferation, high levels of DSB and SSB cause apoptosis, and all other combinations of levels of DSB or SSB induce senescence or transient cycle arrest.

### Model stable states: mutant cases

We briefly present the results obtained for a series of *in silico *perturbations that correspond to loss-of-function (LoF) or gain-of-function (GoF) experiments. The full set of stable states of each perturbation is listed in the Supplementary Material 1 to this article. Table 3 recapitulates the comparisons between the model outcomes and experimental data for mutant cells that have undergone (or not) DNA damage. In these experiments, DNA damage agents include ionizing radiation or carcinogenic chemicals. We searched for experiments preferentially dealing with human cells, but some mutations were found only in mice. Additionally, when data at the cellular level are lacking, we sometimes refer to the mutant mice phenotype.

**Table 3:** Comparison of model outcomes with experiments for different perturbations.

Loss-Of-Function (LoF)		
**Gene**	**Model outcome**	**Experimental outcome**

Comparison with experiments whose protocol include DNA damage		

p38MAPK	Loss of senescence and of apoptosis	Reduced apoptosis [35]

CHEK2	Loss of senescence	Loss of senescence [37]

ATM	Loss of senescence and of apoptosis	Reduced apoptosis [41]

Comparison with experiments whose protocol does not include DNA damage		

CHEK1	No damage: proliferation With damage: loss of senescence	Apoptosis enhanced [42]

p14ARF	No damage: proliferation With damage: senescence enhanced & loss of apoptosis	**?**

Mdm2	No damage: proliferation With damage: apoptosis	Apoptosis [45]

p16^INK4a^	No damage: proliferation With damage: loss of senescence	Loss of senescence [6,7,21]

p21	No damage: proliferation With damage: loss of senescence	Proliferation [47]

p53	No damage: proliferation With damage: loss of senescence & apoptosis	Proliferation & loss of senescence [48]

ATR	No damage: proliferation With damage: senescence enhanced & loss of apoptosis	**?**

CDC25A	No damage: loss of proliferation With damage: senescence enhanced	**?**

RB1	No damage: proliferation With damage: apoptosis	Apoptosis enhanced [50]

E2F	No damage: loss of proliferation With damage: similar to the wild type	Loss of proliferation [51]

p53 & Mdm2	No damage: proliferation With damage: loss of senescence & apoptosis	Proliferation [45]

**Gain-Of-Function (GoF)**		

**Gene**	**Model outcome**	**Experimental outcome**

Comparison with experiments whose protocol include DNA damage		

CHEK2	Senescence enhanced	Apoptosis & senescence [39,40]

Mdm2	Senescence enhanced & loss of apoptosis	Loss of apoptosis [46]

p21	Similar to the wild type	Loss of proliferation or senescence [43]

Comparison with experiments whose protocol does not include DNA damage		

p38MAPK	No damage: [1,2] loss of proliferation; [3] apoptosis With damage: [1,2] senescence enhanced & loss of apoptosis; [3] apoptosis	Senescence [36]

CHEK1	No damage: loss of proliferation With damage: similar to the wild type	**?**

p14ARF	No damage: proliferation With damage: apoptosis	Apoptosis [43]

p16^INK4a^	No damage: cell cycle arrest with probability ~ 0.9 With damage: [1,2] similar to the wild type; [2] senescence enhanced	Proliferation decreased or senescence [6,7,21]

p53	No damage: [1,2] loss of proliferation; [2] apoptosis With damage: [1] senescence enhanced & loss of apoptosis; [2]: apoptosis	Apoptosis [4,48]

ATM	No damage: [1] loss of proliferation; [2] senescence With damage: [1] loss of senescence & apoptosis; [2] senescence enhanced	**?**

ATR	No damage: [1,2] loss of proliferation With damage: [1] senescence enhanced & loss of apoptosis; [2] loss of senescence & apoptosis enhanced	Senescence [32]

CDC25A	No damage: [1,2] proliferation With damage: [1,2] loss of senescence	Proliferation [49]

RB1	No damage: loss of proliferation With damage: similar to the wild type	Cycle arrest [48]

E2F	No damage: proliferation With damage: apoptosis	Apoptosis [51]

Distinction is made between experiments that include DNA damage or not (see Supplementary material). Question marks indicate cases for which no data were found.

It is worth recalling that with cell culture experiments that are currently available, one cannot know in which checkpoint (G1/S or G2/M) the decision is taken. Furthermore, results only provide the predominant fate among cell populations. Still, our comparisons between experimental observations and the behaviour of our cellular model are qualitatively valid in terms of trend with regard to cell fate. Moreover, we include some predictions of the model that remain to be experimentally tested.

p38MAPK knockout decreases apoptosis in mouse fibroblasts [35] whereas its gain-of-function induces senescence in human fibroblasts [36]. Our model outcomes are compatible with these observations.

CHEK2 loss-of-function simulation abrogates senescence, similar to what is seen in thymocytes [37]. Furthermore, the model predicts that when SSB = 2, senescence is induced, as observed in experiments [38]. CHEK2 gain-of-function expression abrogates proliferation, also in agreement with experiments where apoptosis and senescence are enhanced in human DLD1 and HeLa cells [39,40]. Experiments of ATM knockout report a decrease of apoptosis in human endothelial cells (HUVEC cells) [41] while our model abrogates it.

In our model, CHEK1 loss-of-function abrogates senescence, in contrast with experiments that show an increase of apoptosis [42]. However, this discrepancy is to be expected since, beyond the difficulty of comparing such experimental data with our model, CHEK1 is an essential gene involved in other important functions, including the homologous recombination repair and the regulation of G2/M checkpoint, both not included in our model [42].

Gain-of-function experiments of p14ARF induce an apoptotic phenotype in osteosarcoma cells [43]. Accordingly, our model predicts an enhanced apoptosis in the presence of DNA damage, whereas in the absence of damage, proliferation is preserved.

In single cells and mutant mice, Mdm2 knockout induces an apoptotic phenotype [44], which is obtained by our model in the presence of DNA damage. Importantly, consistent with the experimental literature, the model shows that lethality of Mdm2 knockout can be rescued by deleting p53 [45]. Finally, in agreement with our model, ectopic expression of Mdm2 abrogates apoptosis in mice cells [46].

In the case of p16^INK4a ^loss-of-function, there is no stable senescent state, as observed experimentally in several cell types, in which, in absence as well as in presence of DNA damage, p16^INK4a ^gain-of-function induces arrest and senescence enhancement [4,7,21]. Gain-of-function of p16^INK4a ^(maintained positive, between 1 and 2) displays multi-stability in the absence of DNA damage, with two possible fates (proliferation and cycle arrest). By sampling the state space through 10^4 ^random simulations (see Methods), we obtained that the probability of cycle arrest is >0.90. The model thus reproduces the proliferation decrease, but senescence enhancement is only obtained in the presence of DNA damage, with p16^INK4a ^maintained constant at level 2.

In agreement with the model outcomes, p21 loss-of-function induces proliferation in cancer cell lines, while its ectopic expression abrogates proliferation or induces senescence in human cells and mouse fibroblasts [6,47].

p53 knockout induces proliferation and abrogates senescence in some cell types [7], which is consistent with the model outcomes. Additionally, p53 null mice cells show enhanced proliferation and are tumour prone [48]. p53 gain-of-function in mice cells induces an apoptotic phenotype [48], an outcome also obtained with our model.

Experiments of ATR gain-of-function report an increase in senescence in mouse fibroblasts [32], compatible with our model predictions.

In mice fibroblasts, CDC25A loss-of-function and gain-of-function respectively induce or prevent checkpoint arrest [49]. Accordingly, for CDC25A loss-of-function, the model predicts loss of proliferation in the absence of DNA damage. Interestingly, in the presence of DNA damage, the model predicts senescence enhancement for CDC25A loss-of-function, an outcome previously hypothesized [49].

Tissues of RB1 null mice show increased apoptosis while RB1 gain-of-function decreases proliferation [48,50], both phenotypes are recovered by our model.

The model predicts that E2F loss-of-function abrogates proliferation, in agreement with the observation that human fibroblasts arrest or do not proliferate without E2F [51]. E2F gain-of-function induces apoptosis [51], an outcome reproduced by the model in the presence of DNA damage.

Importantly, our model fairly reproduces the observation that a double knockout of Mdmd2 and p53 rescues lethality of Mdm2 knockout [45].

## Conclusion

In summary, we have defined a precursory logical model for the G1/S checkpoint where ATR and ATM pathways activation by single and double DNA strand breaks is sufficient to determine three different cell fates: cell cycle arrest, apoptosis and senescence. ATM/ATR-p38MAPK pathway in our model regulates the senescent fate decision.

Despite a crude abstraction, our model accounts for most experimental evidences, especially regarding the senescent cell fate. Although this phenotype can depend on the cell type, some mutations affecting this state are observed in numerous cell types and reproduced by our model; for instance, the senescent arrest arises from overexpression of p16^INK4a ^[4,7] and its abrogation from p21 or p16^INK4a ^inactivation [7]. Interestingly, the model predicts that CDC25A knockout enhances senescence, an outcome that could lead to a relevant target for intervention in cancer [49].

At first glance, the model stable states seem to depend on the sole value of p38MAPK (see Figure 2). Indeed, the logical rules defining p38MAPK values perfectly match the dependence of the fates to the levels of DSB and SSB. Obviously, this is not the case: the sole value of p38MAPK is not enough to determine the resulting cellular fate. This is demonstrated through perturbation analyses: p38MAPK loss-of-function leads to cell-cycle arrest phenotypes in addition to the proliferative state; for a mild ectopic expression of p38MAPK, a senescent state appears in addition to the expected cell-cycle arrest outcome; finally, a p38MAPK ectopic expression at level 2 leads to the cell-cycle arrest phenotypes, besides the senescent outcome. It is only when p38MAPK is maintained at its level 3 that we have the sole apoptotic outcome.

Moreover, our model displays neither multi-stability (unless in the case of p16^INK4a ^gain-of-function), nor stable oscillations. This can be explained through the analysis of its regulatory circuits. Indeed, regulatory circuits are known to be responsible for the emergence of dynamical properties; negative circuits (*i.e*. encompassing an odd number of inhibitions) are related to oscillations, whereas positive circuits (with an even number of inhibitions) induce multi-stability [52-54]. The (sole) positive circuit of the network encompasses CDK2CycE, RB1 and E2F and has a functionality context in which the system cannot be maintained. Noteworthy, if p16INK4a is maintained at a positive level (1 or 2), this functionality context is stable when DSB=SSB = 0 and indeed, there is multi-stability under this stress condition, with two stable states differing by their values of the circuit members.

The lack of p53 oscillations that are involved in the choice of cell fate [15] can be viewed as a limitation of the model. These oscillations are driven by the negative circuit (p53, Mdm2), known to produce a p53 oscillatory response to DNA damage (e.g. [55]). A closer look to p53 logical function indicates why this circuit is not functional in our model (*i.e*. does not produce stable oscillations [54,56]). The interaction from Mdm2 to p53 selects p53 levels between 1 and 2 (*i.e*. a total inactivation of p53 does not depend on Mdm2), whereas the threshold of the interaction from p53 to Mdm2 is 1. This points towards a potential revision of the model to account for this oscillatory behaviour.

Future extensions include the consideration of components involved in the G2/M checkpoint in response to DNA damage [2,8]. Moreover, the model could be improved by incorporating NF-KB, involved in inflammatory responses and having an anti-apoptotic function in DNA damage response [4,18]. For simplicity, mitogen-activated protein kinase pathways are partly embodied in p38MAPK in our model. Due to their important role in stress-induced cell fate decisions, it would be valuable to extend our model with this network, *e.g*. relying on the logical model recently published by Grieco *et al*. [20].

## Methods

We relied on the generalised logical formalism, initially proposed by R. Thomas and colleagues [57-59]. Here, we briefly describe this modelling framework, typical model properties and the computational tool used to perform model analysis (see e.g. [52,59] for further detail).

Briefly, a logical model is defined by a regulatory graph, by discrete variables associated with the components and rules specifying the evolution of these variables. Nodes in a regulatory graph represent molecular components (genes, proteins, complexes, etc.), processes or phenomenological events (e.g. proliferation, cell-cycle arrest, stress, etc.). Edges embody regulatory effects (activations or inhibitions). Variables represent activity levels (Boolean or multi-valued). Although Boolean variables (0 or 1) are generally enough, a multi-valued variable can be associated with a component to convey different effects upon its targets.

The level of each component evolution is defined by a logical rule that depends on the regulators of this component. Input components (that are not regulated and that embody extrinsic conditions) are considered constant.

The (discrete) dynamical behaviours of logical models are defined in terms of state transition graphs, where nodes represent states (vectors encompassing the components levels), and arcs represent state transitions. Transitions from a state to its successors are defined by the changes in component levels dictated by the logical rules. Terminal strongly components of state transition graphs correspond to attractors, either stable states or cyclic complex attractors; these are (sets of) state(s) that once reached, cannot be left. In particular, a stable state has no successor state since all component levels are stable.

Several updating schemes can be considered, the most common being the synchronous and the asynchronous schemes [57,59]. For the model presented here that has a unique stable state for each (fixed) combination of DNA damage values, the choice of the updating scheme has no impact, as the system will ultimately reach this sole attractor. This is not the case when multiple attractors exist; in this case the asynchronous update is more biologically founded [57]. We have recently developed means to characterise attractor's reachability (manuscript in preparation). By sampling the state space through adapted Monte Carlo simulations, we can estimate the probability associated to each attractor. We applied this new approach to evaluate the probability associated to the proliferative and cycle-arrest attractors for ectopic expression of p16^INK4a ^(see Results).

The logical framework conveniently supports the qualitative nature of current knowledge of most signalling and regulatory mechanisms. Interestingly, implicit notion of time and asynchronous updating schemes allow the consideration of diverse molecular processes associated with different time scales in a single model, from transcriptional regulation to protein phosphorylation [60]. The logical approach further allows an easy, systematic analysis of perturbations, which amount to keeping a variable to its lowest levels (loss-of-function experiment) of to its positive levels (gain-of-function experiment).

The framework is implemented in the software tool GINsim (http://ginsim.org), which offers a variety of functionalities to analyse logical models [61]. In particular, it provides an efficient determination of the stable states of a model.

Regulatory circuit analysis allows to pinpoint circuits that play a crucial role in the emergence of dynamical properties [53,54,56]; negative circuits (*i.e*. encompassing an odd number of inhibitions) are required for oscillations, whereas positive circuits (encompassing an even number of inhibitions) are required for multi-stability. GINsim provides the functionality context (if not empty) of each circuit. This context defines a region in the state space where the circuit generates the expected property (multi-stability or sustained oscillations) [62,63].

Finally, the model file is made available, together with its documentation in the Supplementary material 2to this article.

## Competing interests

The authors declare that they have no competing interests.

## Authors' contributions

JCMM, CC and CB designed the model. JCMM implemented and analyzed the model. JCMM and CC wrote the paper.

## Supplementary Material

Supplementary material 1 Click here for file

Supplementary material 2 Click here for file

## Declarations

The publication costs of this paper were covered by Universidade Federal de Santa Maria.

This article has been published as part of *BMC Genomics *Volume 15 Supplement 7, 2014: Proceedings of the 9th International Conference of the Brazilian Association for Bioinformatics and Computational Biology (X-Meeting 2013). The full contents of the supplement are available online at http://www.biomedcentral.com/bmcgenomics/supplements/15/S7.
