# Supplementary material for: Modelling the onset of senescence at the G1/S cell cycle checkpoint
Source: BMC Genomics. 2014 Oct 27;15(Suppl 7):S7. doi: 10.1186/1471-2164-15-S7-S7 (PMC4243082; doi:10.1186/1471-2164-15-S7-S7)
Supplement: Supplementary material 1 [file 1471-2164-15-S7-S7-S1.pdf]

Supplementary Material

Paper: Modelling the onset of senescence at the G1/S cell cycle checkpoint
Authors: José CM Mombach, Cristhian A Bugs and Claudine Chaouiya

Stable states for the wild type situation and a range of perturbations corresponding to Loss-of-Function (LoF) and Gain-of-Function (GoF) *in silico* experiments (see also Table 3). Results were obtained using GINsim. For a LoF, the component value is constrained to value 0, whereas for a GoF the value is constrained to value 1 (for a Boolean case), or within a range of positive values (indicated between square brackets). States are displayed in the same way as in Figure 2. Each of the 9 combinations of SSB and DSB values (2 right-most columns) leads to a unique stable state denoting a cell fate defined by the values of the outputs (4 left-most columns). Below each table, a short interpretation indicates the main phenotypic changes compared to the wild type situation.

Wild type case

apoptosis	cyclearrest	senescence	proliferation	CHEK1	CHEK2	Mdm2	p14ARF	p53	p21	RB1	p16INK4a	p38MAPK	CDK46CycD	Cdc25A	CDK2CycE	E2F	ATM	ATR	DSB	SSB
			1				1						1	2	1	1				
	1					1		1	1	1	1	1		1				1		1
	1					1		1	1	1	1	1		1			1		1	
	1					1		1	1	1	1	1		1			1	1	1	1
	1			1		1		1	1	1	1	1		1				2		2
	1			1		1		1	1	1	1	1		1			1	2	1	2
	1	1		1	1	1		1	1	1	1	2					2		2	
	1	1		1	1	1		1	1	1	1	2					2	1	2	1
1	1			1	1		1	2	1	1	2	3					2	2	2	2

CHEK2 LoF

apoptosis	cyclearrest	senescence	proliferation	CHEK1	CHEK2	Mdm2	p14ARF	p53	p21	RB1	p16INK4a	p38MAPK	CDK46CycD	Cdc25A	CDK2CycE	E2F	ATM	ATR	DSB	SSB
			1				1						1	2	1	1				
	1					1		1	1	1	1	1		1				1		1
	1					1		1	1	1	1	1		1			1		1	
	1					1		1	1	1	1	1		1			1	1	1	1
	1			1		1		1	1	1	1	1		1				2		2
	1			1		1		1	1	1	1	1		1			1	2	1	2
	1			1		1		1	1	1	1	2		1			2		2	
	1			1		1		1	1	1	1	2		1			2	1	2	1
1	1			1			1	2	1	1	2	3		1			2	2	2	2

Without DNA damage: proliferation
With DNA damage: loss of senescence

CHEK2 GoF

apoptosis	cyclearrest	senescence	proliferation	CHEK1	CHEK2	Mdm2	p14ARF	p53	p21	RB1	p16INK4a	p38MAPK	CDK46CycD	Cdc25A	CDK2CycE	E2F	ATM	ATR	DSB	SSB
	1				1	1		1	1	1				1						
	1				1	1		1	1	1	1	1		1				1		1
	1				1	1		1	1	1	1	1		1			1		1	
	1				1	1		1	1	1	1	1		1			1	1	1	1
	1	1		1	1	1		1	1	1	1	1						2		2
	1	1		1	1	1		1	1	1	1	1					1	2	1	2
	1	1		1	1	1		1	1	1	1	2					2		2	
	1	1		1	1	1		1	1	1	1	2					2	1	2	1
1	1			1	1		1	2	1	1	2	3					2	2	2	2

Without DNA damage: loss of proliferation
With DNA damage: senescence enhanced

CHEK1 LoF

apoptosis	cyclearrest	senescence	proliferation	CHEK1	CHEK2	Mdm2	p14ARF	p53	p21	RB1	p16INK4a	p38MAPK	CDK46CycD	Cdc25A	CDK2CycE	E2F	ATM	ATR	DSB	SSB
			1				1						1	2	1	1				
	1					1		1	1	1	1	1		1				1		1
	1					1		1	1	1	1	1		1				2		2
	1					1		1	1	1	1	1		1			1		1	
	1					1		1	1	1	1	1		1			1	1	1	1
	1					1		1	1	1	1	1		1			1	2	1	2
	1				1	1		1	1	1	1	2		1			2		2	
	1				1	1		1	1	1	1	2		1			2	1	2	1
1	1				1		1	2	1	1	2	3		1			2	2	2	2

Without DNA damage: proliferation
With DNA damage: loss of senescence

CHEK1 GoF

apoptosis	cyclearrest	senescence	proliferation	CHEK1	CHEK2	Mdm2	p14ARF	p53	p21	RB1	p16INK4a	p38MAPK	CDK46CycD	Cdc25A	CDK2CycE	E2F	ATM	ATR	DSB	SSB
	1			1		1		1	1	1				1						
	1			1		1		1	1	1	1	1		1				1		1
	1			1		1		1	1	1	1	1		1				2		2
	1			1		1		1	1	1	1	1		1			1		1	
	1			1		1		1	1	1	1	1		1			1	1	1	1
	1			1		1		1	1	1	1	1		1			1	2	1	2
	1	1		1	1	1		1	1	1	1	2					2		2	
	1	1		1	1	1		1	1	1	1	2					2	1	2	1
1	1			1	1		1	2	1	1	2	3					2	2	2	2

Without DNA damage: loss of proliferation
With DNA damage: similar to the wild type

p14ARF LoF

apoptosis	cyclearrest	senescence	proliferation	CHEK1	CHEK2	Mdm2	p14ARF	p53	p21	RB1	p16INK4a	p38MAPK	CDK46CycD	Cdc25A	CDK2CycE	E2F	ATM	ATR	DSB	SSB
			1										1	2	1	1				
	1					1		1	1	1	1	1		1				1		1
	1					1		1	1	1	1	1		1			1		1	
	1					1		1	1	1	1	1		1			1	1	1	1
	1			1		1		1	1	1	1	1		1				2		2
	1			1		1		1	1	1	1	1		1			1	2	1	2
	1	1		1	1	1		1	1	1	1	2					2		2	
	1	1		1	1	1		1	1	1	1	2					2	1	2	1
	1	1		1	1	1		1	1	1	2	3					2	2	2	2

Without DNA damage: proliferation
With DNA damage: senescence enhanced and loss of apoptosis

p14ARF GoF

apoptosis	cyclearrest	senescence	proliferation	CHEK1	CHEK2	Mdm2	p14ARF	p53	p21	RB1	p16INK4a	p38MAPK	CDK46CycD	Cdc25A	CDK2CycE	E2F	ATM	ATR	DSB	SSB
			1				1						1	2	1	1				
1	1						1	2	1	1	1	1		1				1		1
1	1						1	2	1	1	1	1		1			1		1	
1	1						1	2	1	1	1	1		1			1	1	1	1
1	1			1			1	2	1	1	1	1		1				2		2
1	1			1			1	2	1	1	1	1		1			1	2	1	2
1	1			1	1		1	2	1	1	1	2					2		2	
1	1			1	1		1	2	1	1	1	2					2	1	2	1
1	1			1	1		1	2	1	1	2	3					2	2	2	2

Without DNA damage: proliferation
With DNA damage: apoptosis

p38MAPK LoF

apoptosis	cyclearrest	senescence	proliferation	CHEK1	CHEK2	Mdm2	p14ARF	p53	p21	RB1	p16INK4a	p38MAPK	CDK46CycD	Cdc25A	CDK2CycE	E2F	ATM	ATR	DSB	SSB
			1				1						1	2	1	1				
	1					1		1	1	1				2				1		1
	1					1		1	1	1				2			1		1	
	1					1		1	1	1				2			1	1	1	1
	1			1		1		1	1	1				1				2		2
	1			1		1		1	1	1				1			1	2	1	2
	1			1	1	1		1	1	1				1			2		2	
	1			1	1	1		1	1	1				1			2	1	2	1
	1			1	1	1		1	1	1				1			2	2	2	2

Without DNA damage: proliferation
With DNA damage: loss of senescence and of apoptosis

p38MAPK GoF [1-2]

apoptosis	cyclearrest	senescence	proliferation	CHEK1	CHEK2	Mdm2	p14ARF	p53	p21	RB1	p16INK4a	p38MAPK	CDK46CycD	Cdc25A	CDK2CycE	E2F	ATM	ATR	DSB	SSB
	1									1	1	2		1						
	1					1		1	1	1	1	2		1				1		1
	1					1		1	1	1	1	2		1			1		1	
	1					1		1	1	1	1	2		1			1	1	1	1
	1			1		1		1	1	1	1	2		1				2		2
	1			1		1		1	1	1	1	2		1			1	2	1	2
	1	1		1	1	1		1	1	1	1	2					2		2	
	1	1		1	1	1		1	1	1	1	2					2	1	2	1
	1	1		1	1	1		1	1	1	1	2					2	2	2	2

Without DNA damage: loss of proliferation
With DNA damage: senescence enhanced & loss of apoptosis

p38MAPK GoF [3]

apoptosis	cyclearrest	senescence	proliferation	CHEK1	CHEK2	Mdm2	p14ARF	p53	p21	RB1	p16INK4a	p38MAPK	CDK46CycD	Cdc25A	CDK2CycE	E2F	ATM	ATR	DSB	SSB
1	1						1	2	1	1	2	3		1						
1	1						1	2	1	1	2	3		1				1		1
1	1						1	2	1	1	2	3		1			1		1	
1	1						1	2	1	1	2	3		1			1	1	1	1
1	1			1			1	2	1	1	2	3		1				2		2
1	1			1			1	2	1	1	2	3		1			1	2	1	2
1	1			1	1		1	2	1	1	2	3					2		2	
1	1			1	1		1	2	1	1	2	3					2	1	2	1
1	1			1	1		1	2	1	1	2	3					2	2	2	2

Without DNA damage: apoptosis
With DNA damage: apoptosis

Mdm2 LoF

apoptosis	cyclearrest	senescence	proliferation	CHEK1	CHEK2	Mdm2	p14ARF	p53	p21	RB1	p16INK4a	p38MAPK	CDK46CycD	Cdc25A	CDK2CycE	E2F	ATM	ATR	DSB	SSB
			1				1						1	2	1	1				
1	1							2	1	1	1	1		1				1		1
1	1							2	1	1	1	1		1			1		1	
1	1							2	1	1	1	1		1			1	1	1	1
1	1			1				2	1	1	1	1		1				2		2
1	1			1				2	1	1	1	1		1			1	2	1	2
1	1			1	1			2	1	1	1	2					2		2	
1	1			1	1			2	1	1	1	2					2	1	2	1
1	1			1	1		1	2	1	1	2	3					2	2	2	2

Without DNA damage: proliferation
With DNA damage: apoptosis

Mdm2 GoF

apoptosis	cyclearrest	senescence	proliferation	CHEK1	CHEK2	Mdm2	p14ARF	p53	p21	RB1	p16INK4a	p38MAPK	CDK46CycD	Cdc25A	CDK2CycE	E2F	ATM	ATR	DSB	SSB
			1			1	1						1	2	1	1				
	1					1		1	1	1	1	1		1				1		1
	1					1		1	1	1	1	1		1			1		1	
	1					1		1	1	1	1	1		1			1	1	1	1
	1			1		1		1	1	1	1	1		1				2		2
	1			1		1		1	1	1	1	1		1			1	2	1	2
	1	1		1	1	1		1	1	1	1	2					2		2	
	1	1		1	1	1		1	1	1	1	2					2	1	2	1
	1	1		1	1	1	1	1	1	1	2	3					2	2	2	2

Without DNA damage: proliferation

With DNA damage: senescence enhanced & loss of apoptosis

p16INK4A LoF

apoptosis	cyclearrest	senescence	proliferation	CHEK1	CHEK2	Mdm2	p14ARF	p53	p21	RB1	p16INK4a	p38MAPK	CDK46CycD	Cdc25A	CDK2CycE	E2F	ATM	ATR	DSB	SSB
			1				1						1	2	1	1				
	1					1		1	1	1		1		1				1		1
	1					1		1	1	1		1		1			1		1	
	1					1		1	1	1		1		1			1	1	1	1
	1			1		1		1	1	1		1		1				2		2
	1			1		1		1	1	1		1		1			1	2	1	2
	1			1	1	1		1	1	1		2					2		2	
	1			1	1	1		1	1	1		2					2	1	2	1
1	1			1	1		1	2	1	1		3					2	2	2	2

Without DNA damage: proliferation

With DNA damage: loss of senescence

p16IKN4A GoF [1-2]

apoptosis	cyclearrest	senescence	proliferation	CHEK1	CHEK2	Mdm2	p14ARF	p53	p21	RB1	p16INK4a	p38MAPK	CDK46CycD	Cdc25A	CDK2CycE	E2F	ATM	ATR	DSB	SSB
			1				1				1			2	1	1				
	1									1	1			2						
	1					1		1	1	1	1	1		1				1		1
	1					1		1	1	1	1	1		1			1		1	
	1					1		1	1	1	1	1		1			1	1	1	1
	1			1		1		1	1	1	1	1		1				2		2
	1			1		1		1	1	1	1	1		1			1	2	1	2
	1	1		1	1	1		1	1	1	1	2					2		2	
	1	1		1	1	1		1	1	1	1	2					2	1	2	1
1	1			1	1		1	2	1	1	1	3					2	2	2	2

Without DNA damage: multistability with proliferation (probability 0.09) and cycle arrest (probability 0.91)
With DNA damage: similar to the wild type

p16IKN4A GoF [2]

apoptosis	cyclearrest	senescence	proliferation	CHEK1	CHEK2	Mdm2	p14ARF	p53	p21	RB1	p16INK4a	p38MAPK	CDK46CycD	Cdc25A	CDK2CycE	E2F	ATM	ATR	DSB	SSB
			1				1				2			2	1	1				
	1									1	2			2						
	1	1				1		1	1	1	2	1		1				1		1
	1	1				1		1	1	1	2	1		1			1		1	
	1	1				1		1	1	1	2	1		1			1	1	1	1
	1	1		1		1		1	1	1	2	1		1				2		2
	1	1		1		1		1	1	1	2	1		1			1	2	1	2
	1	1		1	1	1		1	1	1	2	2					2		2	
	1	1		1	1	1		1	1	1	2	2					2	1	2	1
1	1			1	1		1	2	1	1	2	3					2	2	2	2

Without DNA damage: multistability with proliferation (probability 0.09) and cycle arrest (probability 0.91)
With DNA damage: senescence enhanced

p21 LoF

apoptosis	cyclearrest	senescence	proliferation	CHEK1	CHEK2	Mdm2	p14ARF	p53	p21	RB1	p16INK4a	p38MAPK	CDK46CycD	Cdc25A	CDK2CycE	E2F	ATM	ATR	DSB	SSB
			1				1						1	2	1	1				
	1					1		1		1	1	1		1				1		1
	1					1		1		1	1	1		1			1		1	
	1					1		1		1	1	1		1			1	1	1	1
	1			1		1		1		1	1	1		1				2		2
	1			1		1		1		1	1	1		1			1	2	1	2
	1			1	1	1		1		1	1	2					2		2	
	1			1	1	1		1		1	1	2					2	1	2	1
1	1			1	1		1	2		1	2	3					2	2	2	2

Without DNA damage: proliferation
With DNA damage: loss of senescence

p21 GoF

apoptosis	cyclearrest	senescence	proliferation	CHEK1	CHEK2	Mdm2	p14ARF	p53	p21	RB1	p16INK4a	p38MAPK	CDK46CycD	Cdc25A	CDK2CycE	E2F	ATM	ATR	DSB	SSB
	1								1	1				2						
	1					1		1	1	1	1	1		1				1		1
	1					1		1	1	1	1	1		1			1		1	
	1					1		1	1	1	1	1		1			1	1	1	1
	1			1		1		1	1	1	1	1		1				2		2
	1			1		1		1	1	1	1	1		1			1	2	1	2
	1	1		1	1	1		1	1	1	1	2					2		2	
	1	1		1	1	1		1	1	1	1	2					2	1	2	1
1	1			1	1		1	2	1	1	2	3					2	2	2	2

Without DNA damage: loss of proliferation
With DNA damage: similar to the wild type

p53 LoF

apoptosis	cyclearrest	senescence	proliferation	CHEK1	CHEK2	Mdm2	p14ARF	p53	p21	RB1	p16INK4a	p38MAPK	CDK46CycD	Cdc25A	CDK2CycE	E2F	ATM	ATR	DSB	SSB
			1				1						1	2	1	1				
	1									1	1	1		1				1		1
	1									1	1	1		1			1		1	
	1									1	1	1		1			1	1	1	1
	1			1						1	1	1		1				2		2
	1			1						1	1	1		1			1	2	1	2
	1			1	1					1	1	2					2		2	
	1			1	1					1	1	2					2	1	2	1
	1			1	1		1			1	2	3					2	2	2	2

Without DNA damage: proliferation
With DNA damage: loss of senescence and of apoptosis

p53 GoF [1]

apoptosis	cyclearrest	senescence	proliferation	CHEK1	CHEK2	Mdm2	p14ARF	p53	p21	RB1	p16INK4a	p38MAPK	CDK46CycD	Cdc25A	CDK2CycE	E2F	ATM	ATR	DSB	SSB
	1					1		1	1	1				2						
	1					1		1	1	1	1	1		1				1		1
	1					1		1	1	1	1	1		1			1		1	
	1					1		1	1	1	1	1		1			1	1	1	1
	1			1		1		1	1	1	1	1		1				2		2
	1			1		1		1	1	1	1	1		1			1	2	1	2
	1	1		1	1		1	1	1	1	2	3					2	2	2	2
	1	1		1	1	1		1	1	1	1	2					2		2	
	1	1		1	1	1		1	1	1	1	2					2	1	2	1

Without DNA damage: loss of proliferation
With DNA damage: senescence enhanced & loss of apoptosis

p53 GoF [2]

apoptosis	cyclearrest	senescence	proliferation	CHEK1	CHEK2	Mdm2	p14ARF	p53	p21	RB1	p16INK4a	p38MAPK	CDK46CycD	Cdc25A	CDK2CycE	E2F	ATM	ATR	DSB	SSB
1	1					1		2	1	1				2						
1	1					1		2	1	1	1	1		1				1		1
1	1					1		2	1	1	1	1		1			1		1	
1	1					1		2	1	1	1	1		1			1	1	1	1
1	1			1		1		2	1	1	1	1		1				2		2
1	1			1		1		2	1	1	1	1		1			1	2	1	2
1	1			1	1		1	2	1	1	2	3					2	2	2	2
1	1			1	1	1		2	1	1	1	2					2		2	
1	1			1	1	1		2	1	1	1	2					2	1	2	1

Without DNA damage: apoptosis
With DNA damage: apoptosis

ATM LoF

apoptosis	cyclearrest	senescence	proliferation	CHEK1	CHEK2	Mdm2	p14ARF	p53	p21	RB1	p16INK4a	p38MAPK	CDK46CycD	Cdc25A	CDK2CycE	E2F	ATM	ATR	DSB	SSB
			1				1						1	2	1	1			*	
	1					1		1	1	1	1	1		1				1	*	1
	1			1		1		1	1	1	1	1		1				2	*	2

Without DNA damage: proliferation
With DNA damage: loss of senescence and of apoptosis

ATM GoF [1]

apoptosis	cyclearrest	senescence	proliferation	CHEK1	CHEK2	Mdm2	p14ARF	p53	p21	RB1	p16INK4a	p38MAPK	CDK46CycD	Cdc25A	CDK2CycE	E2F	ATM	ATR	DSB	SSB
	1					1		1	1	1	1	1		1			1		*	
	1					1		1	1	1	1	1		1			1	1	*	1
	1			1		1		1	1	1	1	1		1			1	2	*	2

Without DNA damage: loss of proliferation
With DNA damage: loss of senescence and of apoptosis

ATM GoF [2]

apoptosis	cyclearrest	senescence	proliferation	CHEK1	CHEK2	Mdm2	p14ARF	p53	p21	RB1	p16INK4a	p38MAPK	CDK46CycD	Cdc25A	CDK2CycE	E2F	ATM	ATR	DSB	SSB
	1	1		1	1	1		1	1	1	1	2					2		*	
	1	1		1	1	1		1	1	1	1	2					2	1	*	1
1	1			1	1		1	2	1	1	2	3					2	2	*	2

Without DNA damage: senescence
With DNA damage: senescence enhanced

ATR LoF

apoptosis	cyclearrest	senescence	proliferation	CHEK1	CHEK2	Mdm2	p14ARF	p53	p21	RB1	p16INK4a	p38MAPK	CDK46CycD	Cdc25A	CDK2CycE	E2F	ATM	ATR	DSB	SSB
			1				1						1	2	1	1				*
	1					1		1	1	1	1	1		1			1		1	*
	1	1		1	1	1		1	1	1	1	2					2		2	*

Without DNA damage: proliferation
With DNA damage: senescence enhanced & loss of apoptosis

ATR GoF [1]

apoptosis	cyclearrest	senescence	proliferation	CHEK1	CHEK2	Mdm2	p14ARF	p53	p21	RB1	p16INK4a	p38MAPK	CDK46CycD	Cdc25A	CDK2CycE	E2F	ATM	ATR	DSB	SSB
	1					1		1	1	1	1	1		1				1		*
	1					1		1	1	1	1	1		1			1	1	1	*
	1	1		1	1	1		1	1	1	1	2					2	1	2	*

Without DNA damage: loss of proliferation
With DNA damage: senescence enhanced & loss of apoptosis

ATR GoF [2]

apoptosis	cyclearrest	senescence	proliferation	CHEK1	CHEK2	Mdm2	p14ARF	p53	p21	RB1	p16INK4a	p38MAPK	CDK46CycD	Cdc25A	CDK2CycE	E2F	ATM	ATR	DSB	SSB
	1			1		1		1	1	1	1	1		1				2		*
	1			1		1		1	1	1	1	1		1			1	2	1	*
1	1			1	1		1	2	1	1	2	3					2	2	2	*

Without DNA damage: loss of proliferation
With DNA damage: loss of senescence & apoptosis enhanced

RB1 LoF

apoptosis	cyclearrest	senescence	proliferation	CHEK1	CHEK2	Mdm2	p14ARF	p53	p21	RB1	p16INK4a	p38MAPK	CDK46CycD	Cdc25A	CDK2CycE	E2F	ATM	ATR	DSB	SSB
			1				1						1	2	1	1				
1	1						1	2	1		1	1		1		1		1		1
1	1						1	2	1		1	1		1		1	1		1	
1	1						1	2	1		1	1		1		1	1	1	1	1
1	1			1			1	2	1		1	1		1		1		2		2
1	1			1			1	2	1		1	1		1		1	1	2	1	2
1	1			1	1		1	2	1		1	2				1	2		2	
1	1			1	1		1	2	1		1	2				1	2	1	2	1
1	1			1	1		1	2	1		2	3				1	2	2	2	2

Without DNA damage: proliferation
With DNA damage: apoptosis

RB1 GoF

apoptosis	cyclearrest	senescence	proliferation	CHEK1	CHEK2	Mdm2	p14ARF	p53	p21	RB1	p16INK4a	p38MAPK	CDK46CycD	Cdc25A	CDK2CycE	E2F	ATM	ATR	DSB	SSB
	1									1			1	2						
	1					1		1	1	1	1	1		1				1		1
	1					1		1	1	1	1	1		1			1		1	
	1					1		1	1	1	1	1		1			1	1	1	1
	1			1		1		1	1	1	1	1		1				2		2
	1			1		1		1	1	1	1	1		1			1	2	1	2
	1	1		1	1	1		1	1	1	1	2					2		2	
	1	1		1	1	1		1	1	1	1	2					2	1	2	1
1	1			1	1		1	2	1	1	2	3					2	2	2	2

Without DNA damage: loss of proliferation
With DNA damage: similar to the wild type

CDC25A LoF

apoptosis	cyclearrest	senescence	proliferation	CHEK1	CHEK2	Mdm2	p14ARF	p53	p21	RB1	p16INK4a	p38MAPK	CDK46CycD	Cdc25A	CDK2CycE	E2F	ATM	ATR	DSB	SSB
	1									1										
	1	1				1		1	1	1	1	1						1		1
	1	1				1		1	1	1	1	1					1		1	
	1	1				1		1	1	1	1	1					1	1	1	1
	1	1		1		1		1	1	1	1	1						2		2
	1	1		1		1		1	1	1	1	1					1	2	1	2
	1	1		1	1	1		1	1	1	1	2					2		2	
	1	1		1	1	1		1	1	1	1	2					2	1	2	1
1	1			1	1		1	2	1	1	2	3					2	2	2	2

Without DNA damage: loss of proliferation
With DNA damage: senescence enhanced

CDC25A GoF [1-2]

apoptosis	cyclearrest	senescence	proliferation	CHEK1	CHEK2	Mdm2	p14ARF	p53	p21	RB1	p16INK4a	p38MAPK	CDK46CycD	Cdc25A	CDK2CycE	E2F	ATM	ATR	DSB	SSB
			1				1						1	2	1	1				
	1					1		1	1	1	1	1		1				1		1
	1					1		1	1	1	1	1		1			1		1	
	1					1		1	1	1	1	1		1			1	1	1	1
	1			1		1		1	1	1	1	1		1				2		2
	1			1		1		1	1	1	1	1		1			1	2	1	2
	1			1	1	1		1	1	1	1	2		1			2		2	
	1			1	1	1		1	1	1	1	2		1			2	1	2	1
1	1			1	1		1	2	1	1	2	3		1			2	2	2	2

Without DNA damage: proliferation
With DNA damage: loss of senescence

E2F LoF

apoptosis	cyclearrest	senescence	proliferation	CHEK1	CHEK2	Mdm2	p14ARF	p53	p21	RB1	p16INK4a	p38MAPK	CDK46CycD	Cdc25A	CDK2CycE	E2F	ATM	ATR	DSB	SSB
	1												1	2						
	1					1		1	1	1	1	1		1				1		1
	1					1		1	1	1	1	1		1			1		1	
	1					1		1	1	1	1	1		1			1	1	1	1
	1			1		1		1	1	1	1	1		1				2		2
	1			1		1		1	1	1	1	1		1			1	2	1	2
	1	1		1	1	1		1	1	1	1	2					2		2	
	1	1		1	1	1		1	1	1	1	2					2	1	2	1
1	1			1	1		1	2	1	1	2	3					2	2	2	2

Without DNA damage: loss of proliferation
With DNA damage: similar to the wild type

E2F GoF

apoptosis	cyclearrest	senescence	proliferation	CHEK1	CHEK2	Mdm2	p14ARF	p53	p21	RB1	p16INK4a	p38MAPK	CDK46CycD	Cdc25A	CDK2CycE	E2F	ATM	ATR	DSB	SSB
			1				1						1	2	1	1				
1	1						1	2	1	1	1	1		1		1		1		1
1	1						1	2	1	1	1	1		1		1	1		1	
1	1						1	2	1	1	1	1		1		1	1	1	1	1
1	1			1			1	2	1	1	1	1		1		1		2		2
1	1			1			1	2	1	1	1	1		1		1	1	2	1	2
1	1			1	1		1	2	1	1	1	2				1	2		2	
1	1			1	1		1	2	1	1	1	2				1	2	1	2	1
1	1			1	1		1	2	1	1	2	3				1	2	2	2	2

Without DNA damage: proliferation
With DNA damage: apoptosis

p53 and Mdm2 LoF (double mutant)

apoptosis	cyclearrest	senescence	proliferation	CHEK1	CHEK2	Mdm2	p14ARF	p53	p21	RB1	p16INK4a	p38MAPK	CDK46CycD	Cdc25A	CDK2CycE	E2F	ATM	ATR	DSB	SSB
			1				1						1	2	1	1				
	1									1	1	1		1				1		1
	1									1	1	1		1			1		1	
	1									1	1	1		1			1	1	1	1
	1			1						1	1	1		1				2		2
	1			1						1	1	1		1			1	2	1	2
	1			1	1					1	1	2					2		2	
	1			1	1					1	1	2					2	1	2	1
	1			1	1		1			1	2	3					2	2	2	2

Without DNA damage: proliferation
With DNA damage: loss of senescence and of apoptosis
