# Supplementary material for: Modelling the onset of senescence at the G1/S cell cycle checkpoint
Source: BMC Genomics. 2014 Oct 27;15(Suppl 7):S7. doi: 10.1186/1471-2164-15-S7-S7 (PMC4243082; doi:10.1186/1471-2164-15-S7-S7)
Supplement: Supplementary material 2 [file 1471-2164-15-S7-S7-S2.pdf]

Description of the model: "Modelling the onset of senescence at the G1/S cell cycle checkpoint".

Annotation

Model of cell-fate decision upon DNA damage involving the p53/p21 and p38MAPK/p16 pathways affecting G1/S checkpoint.

Nodes

ID	Val	Logical function	Comment
apoptosis	1	• p53:2	Kracikova M, Akiri G, George A, Sachidanandam R, Aaronson SA: A threshold mechanism mediates p53 cell fate decision between growth arrest and apoptosis. Cell Death Differ. 2013, 20:576–588.
cyclearrest	1	• !CDK2CycE	MOLECULAR MECHANISMS OF MAMMALIAN DNA REPAIR AND THE DNA DAMAGE CHECKPOINTS. Aziz Sancar, Laura A. Lindsey-Boltz,<br>Keziban Unsal-Kaçmaz, and Stuart Linn, Annu. Rev. Biochem. 2004. 73:39–85<br>doi:10.1146/annurev.biochem.73.011303.073723
senescence	1	• (p16INK4a:1 & p21 & !p53:2 & !Cdc25A) | (p16INK4a:2 & p21 & !p53:2 & !Cdc25A:2)	Kuilman T, Michaloglou C, Mooi WJ, Peeper DS: The essence of senescence. Genes Dev. 2010, 24:2463–2479.<br><br>Campisi J: Aging, cellular senescence, and cancer. Annu. Rev. Physiol. 2013, 75:685–705.<br><br>Medema RH, Macůrek L: Checkpoint control and cancer. Oncogene 2012, 31:2601–2613.<br><br>Lanigan F, Geraghty JG, Bracken AP: Transcriptional regulation of cellular senescence. Oncogene 2011, 30:2901–2911.<br>**Regulator** – **Comment**<br>p16INK4a – Coppé J-P, Rodier F, Patil CK, Freund A, Desprez P-Y, Campisi J: Tumor suppressor and aging biomarker p16(INK4a) induces cellular senescence without the associated inflammatory secretory phenotype. J. Biol. Chem. 2011, 286:36396–36403.
proliferation	1	• CDK2CycE	MOLECULAR MECHANISMS OF MAMMALIAN DNA REPAIR AND THE DNA DAMAGE CHECKPOINTS. Aziz Sancar, Laura A. Lindsey-Boltz,<br>Keziban Unsal-Kaçmaz, and Stuart Linn, Annu. Rev. Biochem. 2004. 73:39–85<br>doi:10.1146/annurev.biochem.73.011303.073723

<table>
<tr><td>CHEK1</td><td>1</td><td>ATR:2 | ATM:2</td><td>Checkpoint kinase 1
<table>
<tr><th>Regulator</th><th>Comment</th></tr>
<tr><td>ATM</td><td>ATM and ATR Substrate Analysis Reveals Extensive Protein Networks Responsive to DNA Damage
Shuhei Matsuoka et al., Science 25 May 2007: Vol. 316 no. 5828 pp. 1160-1166
DOI: 10.1126/science.1140321</td></tr>
<tr><td>ATR</td><td>MOLECULAR MECHANISMS OF MAMMALIAN DNA REPAIR AND THE DNA DAMAGE CHECKPOINTS. Aziz Sancar, Laura A. Lindsey-Boltz,
Keziban Unsal-Kaçmaz, and Stuart Linn, Annu. Rev. Biochem. 2004. 73:39–85 doi:10.1146/annurev.biochem.73.011303.073723</td></tr>
</table></td></tr>
<tr><td>CHEK2</td><td>1</td><td>ATM:2</td><td>http://www.ncbi.nlm.nih.gov/gene/11200
checkpoint kinase 2
<table>
<tr><th>Regulator</th><th>Comment</th></tr>
<tr><td>ATM</td><td>MOLECULAR MECHANISMS OF MAMMALIAN DNA REPAIR AND THE DNA DAMAGE CHECKPOINTS. Aziz Sancar, Laura A. Lindsey-Boltz,
Keziban Unsal-Kaçmaz, and Stuart Linn, Annu. Rev. Biochem. 2004. 73:39–85 doi:10.1146/annurev.biochem.73.011303.073723</td></tr>
</table></td></tr>
<tr><td>Mdm2</td><td>1</td><td>p53:1 & !p14ARF</td><td>http://www.ncbi.nlm.nih.gov/gene/4193
E3 ubiquitin protein ligase homolog
<table>
<tr><th>Regulator</th><th>Comment</th></tr>
<tr><td>p14ARF</td><td>Stott FJ: The alternative product from the human CDKN2A locus, p14ARF, participates in a regulatory feedback loop with p53 and MDM2. The EMBO Journal 1998, 17:5001–5014.</td></tr>
<tr><td>p53</td><td>Stott FJ: The alternative product from the human CDKN2A locus, p14ARF, participates in a regulatory feedback loop with p53 and MDM2. The EMBO Journal 1998, 17:5001–5014.</td></tr>
</table></td></tr>
<tr><td>p14ARF</td><td>1</td><td>p38MAPK | E2F</td><td>http://www.ncbi.nlm.nih.gov/gene/1029 Transcrip variant of CDKN2A:
This ARF product functions as a stabilizer of the tumor suppressor protein p53 as it can interact with, and sequester, the E3 ubiquitin-protein ligase MDM2, a protein responsible for the degradation of p53. In spite of the structural and functional differences, the CDK inhibitor isoforms and the ARF product encoded by this gene, through the regulatory roles of CDK4 and p53 in cell cycle G1 progression, share a common functionality in cell cycle G1 control.
<table>
<tr><th>Regulator</th><th>Comment</th></tr>
<tr><td>E2F</td><td>Balancing the decision of cell proliferation and cell fate. Timothy C. Hallstrom1and Joseph R. Nevins, Cell Cycle 8:4, 532-535; 2009</td></tr>
</table></td></tr>
</table>

<table>
<tr><td></td><td></td><td></td><td><table><tr><td>p38MAPK</td><td>Bulavin DV, Phillips C, Nannenga B, Timofeev O, Donehower LA, Anderson CW, Appella E, Fornace AJ: Inactivation of the Wip1 phosphatase inhibits mammary tumorigenesis through p38 MAPK–mediated activation of the p16Ink4a-p19Arf pathway. Nature Genetics 2004, 36:343–350.</td></tr></table></td></tr>
<tr><td rowspan="2">p53</td><td>1</td><td>• Mdm2 & (p38MAPK | ATR | ATM | CHEK1 | CHEK2)</td><td rowspan="2">• http://www.ncbi.nlm.nih.gov/gene/7157
Tumor supressor protein p53<table><tr><th>Regulator</th><th>Comment</th></tr><tr><td>CHEK1</td><td>MOLECULAR MECHANISMS OF MAMMALIAN DNA REPAIR AND THE DNA DAMAGE CHECKPOINTS. Aziz Sancar, Laura A. Lindsey-Boltz,
Keziban Unsal-Kaçmaz, and Stuart Linn, Annu. Rev. Biochem. 2004. 73:39–85 doi:10.1146/annurev.biochem.73.011303.073723</td></tr><tr><td>ATM</td><td>MOLECULAR MECHANISMS OF MAMMALIAN DNA REPAIR AND THE DNA DAMAGE CHECKPOINTS. Aziz Sancar, Laura A. Lindsey-Boltz,
Keziban Unsal-Kaçmaz, and Stuart Linn, Annu. Rev. Biochem. 2004. 73:39–85 doi:10.1146/annurev.biochem.73.011303.073723</td></tr><tr><td>Mdm2</td><td>Stott FJ: The alternative product from the human CDKN2A locus, p14ARF, participates in a regulatory feedback loop with p53 and MDM2. The EMBO Journal 1998, 17:5001–5014.</td></tr><tr><td>CHEK2</td><td>MOLECULAR MECHANISMS OF MAMMALIAN DNA REPAIR AND THE DNA DAMAGE CHECKPOINTS. Aziz Sancar, Laura A. Lindsey-Boltz,
Keziban Unsal-Kaçmaz, and Stuart Linn, Annu. Rev. Biochem. 2004. 73:39–85 doi:10.1146/annurev.biochem.73.011303.073723</td></tr><tr><td>ATR</td><td>MOLECULAR MECHANISMS OF MAMMALIAN DNA REPAIR AND THE DNA DAMAGE CHECKPOINTS. Aziz Sancar, Laura A. Lindsey-Boltz,
Keziban Unsal-Kaçmaz, and Stuart Linn, Annu. Rev. Biochem. 2004. 73:39–85 doi:10.1146/annurev.biochem.73.011303.073723</td></tr><tr><td>p38MAPK</td><td>Kuilman T, Michaloglou C, Mooi WJ, Peeper DS: The essence of senescence. Genes Dev. 2010, 24:2463–2479.</td></tr></table></td></tr>
<tr><td>2</td><td>• !Mdm2 & (p38MAPK | ATR | ATM | CHEK1 | CHEK2)</td></tr>
<tr><td>p21</td><td>1</td><td>• p53:1</td><td>• http://www.ncbi.nlm.nih.gov/gene/1026
cyclin-dependent kinase inhibitor 1A (p21, Cip1)<table><tr><th>Regulator</th><th>Comment</th></tr><tr><td>p53</td><td>MOLECULAR MECHANISMS OF MAMMALIAN DNA REPAIR AND THE DNA DAMAGE CHECKPOINTS. Aziz Sancar, Laura A. Lindsey-Boltz, Keziban Unsal-Kaçmaz, and Stuart Linn, Annu. Rev. Biochem. 2004. 73:39–85</td></tr></table></td></tr>
</table>

			doi:10.1146/annurev.biochem.73.011303.073723
RB1	1	• !CDK46CycD & !CDK2CycE	• http://www.ncbi.nlm.nih.gov/gene/5925<br>retinoblastoma 1<br>**Regulator** — **Comment**<br>CDK2CycE — MOLECULAR MECHANISMS OF MAMMALIAN DNA REPAIR AND THE DNA DAMAGE CHECKPOINTS. Aziz Sancar, Laura A. Lindsey-Boltz, Keziban Unsal-Kaçmaz, and Stuart Linn, Annu. Rev. Biochem. 2004. 73:39–85 doi:10.1146/annurev.biochem.73.011303.073723
p16INK4a	1	• p38MAPK:1	• http://www.ncbi.nlm.nih.gov/gene/1029<br>Transcript variant of CDKN2A:<br>Cyclin-dependent kinase inhibitor 2A<br><br>Coppé J-P, Rodier F, Patil CK, Freund A, Desprez P-Y, Campisi J: Tumor suppressor and aging biomarker p16(INK4a) induces cellular senescence without the associated inflammatory secretory phenotype. J. Biol. Chem. 2011, 286:36396–36403.<br>**Regulator** — **Comment**<br>p38MAPK — Bulavin DV, Phillips C, Nannenga B, Timofeev O, Donehower LA, Anderson CW, Appella E, Fornace AJ: Inactivation of the Wip1 phosphatase inhibits mammary tumorigenesis through p38 MAPK–mediated activation of the p16Ink4a-p19Arf pathway. Nature Genetics 2004, 36:343–350.
	2	• p38MAPK:3	
p38MAPK	1	• (ATM:1 | ATR:1 | ATR:2) & !ATM:2	• http://www.ncbi.nlm.nih.gov/gene/1432<br>Mitogen activated protein kinase 14<br><br>Lafarga V, Cuadrado A, Lopez de Silanes I, Bengoechea R, Fernandez-Capetillo O, Nebreda AR: p38 Mitogen-Activated Protein Kinase- and HuR-Dependent Stabilization of p21Cip1 mRNA Mediates the G1/S Checkpoint. Molecular and Cellular Biology 2009, 29:4341–4351.<br>**Regulator** — **Comment**<br>ATM — Medema RH, Macůrek L: Checkpoint control and cancer. Oncogene 2012, 31:2601–2613.<br>ATR — Medema RH, Macůrek L: Checkpoint control and cancer. Oncogene 2012, 31:2601–2613.
	2	• !ATR:2 & ATM:2	
	3	• ATM:2 & ATR:2	
CDK46CycD	1	• !p16INK4a & !p21 & Cdc25A	cyclin-dependent kinase 4, 6 and Cyclin D complex<br>**Regulator** — **Comment**<br>p21 — MOLECULAR MECHANISMS OF MAMMALIAN DNA REPAIR AND THE DNA DAMAGE CHECKPOINTS. Aziz Sancar, Laura A. Lindsey-Boltz, Keziban Unsal-Kaçmaz, and Stuart Linn, Annu. Rev. Biochem. 2004. 73:39–85

			doi:10.1146/annurev.biochem.73.011303.073723<br>p16INK4a — Lanigan F, Geraghty JG, Bracken AP: Transcriptional regulation of cellular senescence. Oncogene 2011, 30:2901–2911.
Cdc25A	1	• (p38MAPK | CHEK1 | CHEK2) & !(p38MAPK & CHEK1 & CHEK2)	• http://www.ncbi.nlm.nih.gov/gene/993<br>cell division cycle 25A<br>**Regulator** — **Comment**<br>CHEK1 — MOLECULAR MECHANISMS OF MAMMALIAN DNA REPAIR AND THE DNA DAMAGE CHECKPOINTS. Aziz Sancar, Laura A. Lindsey-Boltz, Keziban Unsal-Kaçmaz, and Stuart Linn, Annu. Rev. Biochem. 2004. 73:39–85 doi:10.1146/annurev.biochem.73.011303.073723<br>CHEK2 — MOLECULAR MECHANISMS OF MAMMALIAN DNA REPAIR AND THE DNA DAMAGE CHECKPOINTS. Aziz Sancar, Laura A. Lindsey-Boltz, Keziban Unsal-Kaçmaz, and Stuart Linn, Annu. Rev. Biochem. 2004. 73:39–85 doi:10.1146/annurev.biochem.73.011303.073723<br>p38MAPK — Bulavin DV, Phillips C, Nannenga B, Timofeev O, Donehower LA, Anderson CW, Appella E, Fornace AJ: Inactivation of the Wip1 phosphatase inhibits mammary tumorigenesis through p38 MAPK–mediated activation of the p16Ink4a-p19Arf pathway. Nature Genetics 2004, 36:343–350.
	2	• !p38MAPK & !CHEK2 & !CHEK1	
CDK2CycE	1	• !p21 & E2F & Cdc25A	cyclin-dependent kinase 2 and Cyclin E complex<br>**Regulator** — **Comment**<br>E2F — MOLECULAR MECHANISMS OF MAMMALIAN DNA REPAIR AND THE DNA DAMAGE CHECKPOINTS. Aziz Sancar, Laura A. Lindsey-Boltz, Keziban Unsal-Kaçmaz, and Stuart Linn, Annu. Rev. Biochem. 2004. 73:39–85 doi:10.1146/annurev.biochem.73.011303.073723<br>Cdc25A — MOLECULAR MECHANISMS OF MAMMALIAN DNA REPAIR AND THE DNA DAMAGE CHECKPOINTS. Aziz Sancar, Laura A. Lindsey-Boltz, Keziban Unsal-Kaçmaz, and Stuart Linn, Annu. Rev. Biochem. 2004. 73:39–85 doi:10.1146/annurev.biochem.73.011303.073723<br>p21 — MOLECULAR MECHANISMS OF MAMMALIAN DNA REPAIR AND THE DNA DAMAGE CHECKPOINTS. Aziz Sancar, Laura A. Lindsey-Boltz, Keziban Unsal-Kaçmaz, and Stuart Linn, Annu. Rev. Biochem. 2004. 73:39–85 doi:10.1146/annurev.biochem.73.011303.073723
E2F	1	• !RB1	• http://www.ncbi.nlm.nih.gov/gene/1869

			E2F transcription factor 1<br>**Regulator** / **Comment**<br>RB1 / The regulation of E2F by pRB-family proteins. Nicholas Dyson, Genes Dev. 1998 12: 2245-2262. 10.1101/gad.12.15.2245
ATM	1	• DSB:1	• http://www.ncbi.nlm.nih.gov/gene/472<br>ATM ataxia telangiectasia mutated
	2	• DSB:2	
ATR	1	• SSB:1	• http://genesdev.cshlp.org/content/22/11/1478.long<br>• http://www.ncbi.nlm.nih.gov/gene/545<br>Ataxia telangiectasia and Rad3 related
	2	• SSB:2	
DSB		no function	• http://jcs.biologists.org/content/early/2010/11/30/jcs.071340<br>DNA double-strand break.<br>States:<br>0: no DSB<br>1: repairable DSB<br>2: irrepairable DSB<br><br>MOLECULAR MECHANISMS OF MAMMALIAN DNA REPAIR AND THE DNA DAMAGE CHECKPOINTS. Aziz Sancar, Laura A. Lindsey-Boltz, Keziban Unsal-Kaçmaz, and Stuart Linn, Annu. Rev. Biochem. 2004. 73:39–85 doi:10.1146/annurev.biochem.73.011303.073723
SSB		no function	DNA single-strand break.<br>States:<br>0: no SSB<br>1: repairable SSB<br>2: irrepairable SSB<br><br>MOLECULAR MECHANISMS OF MAMMALIAN DNA REPAIR AND THE DNA DAMAGE CHECKPOINTS. Aziz Sancar, Laura A. Lindsey-Boltz, Keziban Unsal-Kaçmaz, and Stuart Linn, Annu. Rev. Biochem. 2004. 73:39–85 doi:10.1146/annurev.biochem.73.011303.073723
